# Computational Method for Estimating DNA Copy Numbers in Normal Samples, Cancer Cell Lines, and Solid Tumors Using Array Comparative Genomic Hybridization

**DOI:** 10.1155/2010/386870

**Published:** 2010-07-08

**Authors:** Victor Abkevich, Diana Iliev, Kirsten M. Timms, Thanh Tran, Mark Skolnick, Jerry S. Lanchbury, Alexander Gutin

**Affiliations:** Myriad Genetics Inc., 320 Wakara Way, Salt Lake City, UT 84108, USA

## Abstract

Genomic copy number variations are a typical feature of cancer. These variations may influence cancer outcomes as well as effectiveness of treatment. There are many computational methods developed to detect regions with deletions and amplifications without estimating actual copy numbers (CN) in these regions. We have developed a computational method capable of detecting regions with deletions and amplifications as well as estimating actual copy numbers in these regions. The method is based on determining how signal intensity from different probes is related to CN, taking into account changes in the total genome size, and incorporating into analysis contamination of the solid tumors with benign tissue. Hidden Markov Model is used to obtain the most likely CN solution. The method has been implemented for Affymetrix 500K GeneChip arrays and Agilent 244K oligonucleotide arrays. The results of CN analysis for normal cell lines, cancer cell lines, and tumor samples are presented. The method is capable of detecting copy number alterations in tumor samples with up to 80% contamination with benign tissue. Analysis of 178 cancer cell lines reveals multiple regions of common homozygous deletions and strong amplifications encompassing known tumor suppressor genes and oncogenes as well as novel cancer related genes.

## 1. Introduction

Genomes of cancer cells are known to harbor multiple regions of deletions and amplifications. Many of these CN alterations are probably random but some accelerate cell growth and suppress apoptosis. For example, homozygous deletions lead to inactivation of such tumor suppressor genes as RB1 [1], p16 [2], and PTEN [3], while amplifications lead to activation of such oncogenes as MYC [4], EGFR [5], and ERBB2 [6]. Moreover, it has been shown that heterogeneity of cancer outcomes [7] and sensitivity to chemotherapy can be at least, in part, explained by CN changes in primary tumor [8, 9]. Thus it is of great importance to identify CN variations frequent for a specific tumor type or present in a specific sample. Array comparative genomic hybridization is a method designed to detect such genomic region alterations [10]. The development of high-density arrays as well as advances in data analysis have greatly improved our ability to determine CN regions [11, 12]. Different methods of analysis have been reviewed by Chari et al. [13].

Many of these computational methods detect regions with deletions and amplifications without estimating the actual CN in these regions. For example, these methods do not clearly distinguish between homozygous (CN = 0) and heterozygous (CN = 1) deletions which may have significantly different effects on cancer development; losing just one of two copies of a gene should not have as drastic of an effect as losing both copies. In fact, heterozygous deletions are very common and affect large regions including chromosomal arms and whole chromosomes. In contrast, homozygous deletions are much less common and usually affect small regions with only a few genes deleted. Similarly, it is important to be able to distinguish between low and high copy amplifications. Due to their overall genomic instability, cancer cells might accumulate multiple random CN changes which do not contribute to cancer development. Accordingly, one should expect that only regions with very high amplification, 10 to 100 copies, are regions that are likely to encompass oncogenes such as MYC, EGFR, and ERBB2.

In this study we attempted to develop a method to estimate CN values for normal samples, cell lines, and solid tumors. To be able to do this we had to determine how signal intensity from different probes is related to CN, how to take into account change of the genome size in cancer cell lines and solid tumors which result from somatic alterations, and finally how to incorporate into the analysis contamination of the solid tumors with benign tissue. The last factor is of particular importance as solid tumor samples are strongly contaminated with noncancerous cells carrying DNA without somatic alterations. Existing dissection techniques can reduce this contamination but cannot eliminate it entirely. For example, it is practically impossible to eliminate immune cell infiltration of tumors. In our experience, even ovarian tumors which are considered less affected by noncancerous contamination than other tumors often contain more than 50% normal, noncancerous, DNA. Such strong contamination presents a significant problem for estimating CN. For example, if in a tumor sample a particular region appears to have one copy, the actual CN in cancerous cells can be either one (CN = 1) if the sample has negligible contamination or zero (CN = 0) if the contamination is about 50%.

## 2. Materials and Methods

### 2.1. Genomic DNA

Frozen tumors were cut into 10 *μ*m sections and macrodissected to minimize contamination with normal tissue. A QIAamp DNA Mini Kit (QIAgen) was used to isolate the DNA as per the manufacturer's protocol with an overnight lysis incubation at 56°C, including the optional RNase A treatment. DNA was quantitated using a Nanodrop spectrophotometer and picogreen.

### 2.2. Affymetrix 500K GeneChip Arrays

The Affymetrix GeneChip Mapping NspI or StyI Assay Kit was used in the generation of biotinylated DNA for Affymetrix Mapping 500K NspI or StyI microarray hybridizations (each assay was prepared separately). Genomic DNA (250 ng) was digested with NspI or StyI restriction enzyme and adaptors were added to restriction fragment ends with T4 DNA ligase. Adaptor-modified samples were PCR amplified using Clontech Titanium Taq which generated an amplified product of average size between 200 and 1,100 bp. Amplification products were purified using a Clontech DNA amplification cleanup kit. 90 *μ*g of purified DNA was fragmented using Affymetrix Fragmentation Reagent. Biotin-labeling of the fragmented sample was accomplished using the GeneChip DNA Labeling Reagent. Biotin-labeled DNA was hybridized on NspI or StyI Affymetrix microarrays at 49°C for 16 to 18 hours in the Affymetrix rotation oven. After hybridization, probe array wash and stain procedures were carried out on the automatic Affymetrix Fluidics Stations as per manufacturer's manual and microarrays were scanned and raw data were collected by Affymetrix GeneChip Scanner 3000.

### 2.3. Quantitative Real-Time PCR

12 cell lines were identified with copy number amplifications detected by microarray spanning the genes CCND1, EGFR, and/or ERBB2. Custom qPCR assays (Applied Biosystems) were designed which would amplify a region of genomic DNA from each of these genes, and from 2 genomic regions where the cell lines did not show any copy number changes. These 2 assays were used as calibrators to calculate dCt in subsequent analyses. Genomic DNA from each of the cell lines was diluted to 5 ng/*μ*l. 1 *μ*L genomic DNA, 8 *μ*l H_2_O, 1 *μ*l qPCR assay, and 10 *μ*l TaqMan Universal PCR Master Mix (Applied Biosystems) were combined and cycled in an Applied Biosystems 7900 real-time PCR instrument using the following conditions: 50°C for 2 minutes, 95°C for 10 minutes, 40 cycles of 95°C for 15 seconds, 60°C for 1 minute, hold at 4°C.

### 2.4. Agilent 244K CGH Arrays

0.5–3 *μ*g test and reference (Promega, p/n G152A) genomic DNA samples were simultaneously digested with AluI and RsaI restriction enzymes. Following fragmentation, the DNA samples were labeled using an Agilent Genomic Enzymatic Labeling Kit. The labeling kit uses random primers and the exo-Klenow fragment to label DNA through incorporation of fluorescently labeled nucleotides (Cy3-dUTP or Cy5-dUTP, for test or reference DNA, resp.). Labeled samples were purified by Microcon YM-30 columns or AutoScreen-96A Well plates. The concentration of the purified samples was determined using a NanoDrop ND-1000 spectrophotometer. 

Equal amounts of test and reference fluorescently labeled samples were pooled and heat denatured after being combined with cot-1 DNA, Agilent aCGH blocking agent, and Agilent Hi-RPM hybridization solution. Microarray hybridizations were performed using Agilent SureHyb Hybridization chambers. Hybridization chambers were loaded onto a rotisserie in an Agilent Hybridization Oven and were incubated at 65°C for 40 hours with a rotational speed of 20 rpm. Following incubation, the microarray slide was washed for 5 minutes in aCGH/ChIP-on-chip Agilent Wash Buffer 1 at room temperature and 1 minute in Agilent Wash Buffer 2 at 37°C. Microarray slides were scanned on an Agilent scanner and raw data were collected.

## 3. Results

### 3.1. Combining Signals from Multiple Probes on Affymetrix 500K GeneChip Arrays

The Affymetrix 500k GeneChip array contains 25-mer oligonucleotides distributed over two subarrays, Nsp1 and Sty1 containing 262,264 and 238,304 SNPs, respectively. Each SNP on the array is represented by either six or ten oligonucleotide probe quartets consisting of perfect-match (PM) and mismatch (MM) pairs for both SNP alleles. These probes reside on Nsp and Sty PCR amplicons, which range in size from 100 bp to 1143 bp. For CN analysis the signal intensities from the multiple probes representing a SNP should be optimally combined to generate a single value corresponding to the SNP. 

It has been shown [14] that GC content and length of PCR amplicons affect signal intensities from probes and this effect varies from sample to sample. It also appears that the effect of amplicon size is much stronger than the effect of GC content. Our data (not shown) support this conclusion. Therefore, we did not take GC content into account. However, we made an adjustment for amplicon size. First for each quartet and allele, the intensity *I*_MM_ from the MM probe is subtracted from the intensity *I*_PM_ from the PM probe: Δ*I* = *I*_PM_ − *I*_MM_. Then for a given amplicon size *s,* an average *∆I_s_* over all quartets and alleles for SNPs residing on PCR amplicons of size *s* within a subarray is calculated. Finally, a normalized intensity *I*_*qX*_ = Δ*I*/Δ*I*_*s*_ is calculated for each allele *X* and each quartet *q* where *s* is the size of the corresponding amplicon. 

The next step is to combine signals from both alleles *A* and *B* within a quartet *q* using the following formula:

(1) $$S_{q}=k_{A}I_{qA}+k_{B}I_{qB},$$

where *k*_*A*_ and *k*_*B*_ are normalization parameters. These parameters are introduced to ensure that *S*_*q*_ is close to 2 independent of the genotype (*AA, AB, or BB*) of a sample. The ratio of these parameters, *k*_*A*_/*k*_*B*_, represents uneven allele amplification described previously for SNP arrays [15]. In order to estimate parameters *k*_*A*_ and *k*_*B*_, we have used genotyping data for 48 cell lines provided by Affymetrix. Because these cell lines were collected from noncancerous tissue, we assume that for almost all SNPs copy number will be equal to 2. Therefore, for each SNP we sought to minimize the following expression:

(2) $$\begin{array}{cc} E\left(k_{A},k_{B}\right) & =N_{AA}{\left(k_{A}x_{AA}+k_{B}y_{AA}-2\right)}^{2}\\ & \;+N_{AB}{\left(k_{A}x_{AB}+k_{B}y_{AB}-2\right)}^{2}\\ & \;+N_{BB}{\left(k_{A}x_{BB}+k_{B}y_{BB}-2\right)}^{2}. \end{array}$$

Here *N*_*XX*_ is the number of samples with genotype *XX* for this SNP among 48 samples (*N*_*AA*_ + *N*_*AB*_ + *N*_*BB*_ = 48), while *x*_*XX*_ is the median value of *I*_*qA*_ in samples with genotype *XX* and *y*_*XX*_ is the median value of *I*_*qB*_ in these samples. Minimizing the function *E*(*k*_*A*_, *k*_*B*_) over parameters *k*_*A*_ and *k*_*B*_ results in the following formulae:

(3) $$\begin{array}{c} k_{A}=\frac{2\left(C_{x}C_{yy}-C_{y}C_{xy}\right)}{\left(C_{xx}C_{yy}-C_{xy}C_{xy}\right)},\\ k_{B}=\frac{2\left(C_{y}C_{xx}-C_{x}C_{xy}\right)}{\left(C_{xx}C_{yy}-C_{xy}C_{xy}\right)}. \end{array}$$

where

(4) $$\begin{array}{c} C_{x}=N_{AA}x_{AA}+N_{AB}x_{AB}+N_{BB}x_{BB},\\ C_{y}=N_{AA}y_{AA}+N_{AB}y_{AB}+N_{BB}y_{BB},\\ C_{xy}=N_{AA}x_{AA}y_{AA}+N_{AB}x_{AB}y_{AB}+N_{BB}x_{BB}y_{BB},\\ C_{xx}=N_{AA}x_{AA}x_{AA}+N_{AB}x_{AB}x_{AB}+N_{BB}x_{BB}x_{BB},\\ C_{yy}=N_{AA}y_{AA}y_{AA}+N_{AB}y_{AB}y_{AB}+N_{BB}y_{BB}y_{BB}. \end{array}$$

Using these formulae we calculated parameters *k*_*A*_ and *k*_*B*_ for all SNPs on Affymetrix 500K GeneChip arrays except for 10,841 SNPs for which all 48 cell lines were homozygous. These SNPs were excluded from the analysis. To evaluate the strength of the uneven allele amplification effect we calculated the median of max (*k*_*A*_/*k*_*B*_, *k*_*B*_/*k*_*A*_). The median is about 1.40, meaning that for a typical SNP on Affymetrix 500K GeneChip arrays, signal intensity for one of the alleles is about 40% higher than the signal intensity for the other allele. 

The values *S*_*q*_ for all quartets for SNP *i* are linearly combined with weights reflecting their performance: *S*_*i*_ = ∑_*q*_*w*_*q*_*S*_*q*_. The weights are selected to minimize the variance of *S*_*i*_ under the assumption that the deviations of *S*_*q*_ from 2 are independent for different quartets. Thus, the optimal weights are determined by the following formula:

(5) $$w_{q}=\frac{\left(1/\sigma_{q}^{2}\right)}{\sum_{p}\left(1/\sigma_{p}^{2}\right)},$$

where *σ*_*q*_^2^ is the variance of *S*_*q*_ within 48 cell lines. The estimated variance of *S*_*i*_ is

(6) $$\sigma_{i}^{2}=\frac{1}{\sum_{q}\left(1/\sigma_{q}^{2}\right)}.$$

To evaluate the importance of optimization of weights for different quartets, we can compare *σ*_*i*_^2^ with the corresponding variance resulting from using equal weights for all quartets. The estimate for the latter variance is *s*_*i*_^2^ = (1/*n*_*i*_)∑_*q*_*σ*_*q*_^2^, where *n*_*i*_ is the number of quartets for SNP *i*. Median ratio of the variances, *s*_*i*_^2^/*σ*_*i*_^2^, over all SNPs is about 1.97 which means that for a typical SNP using optimal weights should reduce variance almost twofold.

For each SNP we have calculated *σ*_*i*_^2^ defined as (*S*_*i*_−2)^2^ averaged over 48 cell lines. The median *σ*_*i*_^2^ for all SNPs is 0.0376. We have excluded 1,832 SNPs with *σ*_*i*_^2^ exceeding an arbitrary cutoff 0.25. The important exception of the described method concerns SNPs on the X chromosome (outside the pseudoautosomal region). For these SNPs parameters were determined using only cell lines from females.

Some of the PCR amplicons on the Affymetrix 500K GeneChip array contain more than one SNP. Any CN changes affecting such an amplicon should equally affect all SNPs within the amplicon. Therefore, signals *S*_*i*_ for all SNPs within an amplicon are averaged and assigned to one of the SNPs while the other SNPs are excluded from further analysis. This reduces the number of SNPs by 82,189 leaving 405,706 SNPs for CN analysis.

### 3.2. Relationship between SNP Signal Intensity and CN

**Figure 1:**
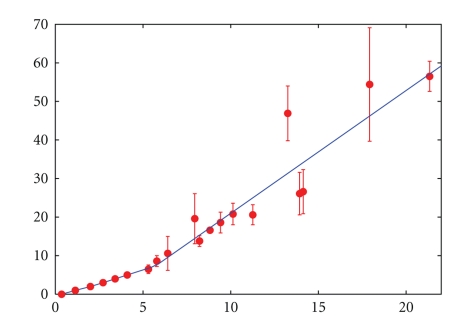
Comparison between median signal intensities calculated using parameters generated in this paper (*y*-axis) and CN estimates obtained in a different way (*x*-axis). For details see Table 1. Blue line represents correlation between median signal intensities and CN values used in this study.

While normalized SNP signal intensities *S*_*i*_ described in the previous section are supposed to generate an average signal of 2.0 for SNPs within regions of genome with CN = 2, one should not expect that the average signal is equal to the actual CN in the regions of the genome where CN is different from two. For example, the slope of the relationship between signal intensity from an array and the amount of DNA in the solution differs from the ideal value 1.0 [16]. Moreover, this relationship might be nonlinear as well. To evaluate this effect for regions of the genome with CN = 1 we used SNPs on the X chromosome (outside the pseudoautosomal regions) for 28 cell lines from males provided by Affymetrix. In addition, we have analyzed cell lines containing different numbers of X chromosomes (all karyotypes in the paper are described accordingly to ISCN 2009 nomenclature [17]): NA04626 (47,XXX), NA01416E (48,XXXX), and NA06061 (49,XXXXX). This allowed us to estimate the relationship between CN and signal intensity up to CN = 5. Finally, we have selected a few cancer cell lines for which it has been reported that certain genes are strongly amplified. These cell lines and a reference sample RS were analyzed by qPCR assays amplifying the corresponding genes as well as a reference gene RG which has 2 copies in these cell lines. CNs for the amplified genes were estimated using the formula CN = 2^(1−*C*_*Tgc*_+*C*_*Trc*_+*C*_*Tgr*_−*C*_*Trr*_)^, where *C*_*Txy*_ is the *C*_*T*_ value for gene *x *(*x* = *g* for an amplified gene and *x* = *r* for the reference gene) in sample *y *(*y* = *c* for a cell line and *y* = *r* for the reference sample). The same cell lines were run on Affymetrix 500K GeneChip arrays. Comparison of the results allowed us to estimate the relationship between CN and signal intensity for high CN values. Results of the experiments are shown in Table 1 and Figure 1. Signal intensity for SNPs in regions with CN > 8 increases roughly linearly with CN. Therefore, for CN *c* the expected signal intensity *S*_0_(*c*) is given by Table 1 for *c* < 8 and *S*_0_(*c*) = 0.314*c* + 3.41 for *c* > 7.

### 3.3. Adjustment for Average CN and Contamination with Normal DNA

As described above, one of the steps in deriving *S*_*i*_ involves normalization by average intensity of other SNPs. This normalization allows accurate estimation of CN for noncancerous samples where most of the genome has two copies. In cancerous samples the average CN, due to amplifications and deletions, can be very different from two and, therefore, additional signal normalization is required. In addition, tumor samples are often contaminated with noncancerous cells with most of the genome having two copies. As a result, the expected signal intensity *S*(*c*) for an SNP within regions with CN *c* is defined by the following equation:

(7) $$\begin{array}{c} S\left(c\right)=\beta \left(S_{0}\left(2\right)\alpha +\left(1-\alpha \right)S_{0}\left(c\right)\right)=\beta \left(2\alpha +\left(1-\alpha \right)S_{0}\left(c\right)\right)\\ . \end{array}$$

Here *β* is a normalization factor to account for change in average CN within the genome of cancerous cells, and *α* is the degree of contamination of tumor samples with normal cells. This equation is applicable to all types of samples: normal noncancerous cells, cancer cell lines, and tumors. However, for cancer cell lines we assume no contamination with noncancerous cells and, therefore, *α* = 0. For noncancerous samples we assume that the average CN is equal to 2.0 and, therefore, *β* = 1 and *α* = 0.

In order to estimate the parameters *α* and *β*, we use the following algorithm. First, for each SNP *i* we calculate smoothed signal *T*_*i*_:

(8) $$\log\;\;\left(T_{i}\right)=\left(\frac{1}{N}\right)\underset{j}{\sum }\log\;\;\left(S_{j}\right),$$

where the sum is taken over *N* = 101 SNPs surrounding SNP *i*  (*j* = *i* − 50,…, *i* + 50). Then we calculate the histogram *H*(*T*) of the smoothed signals with bin size of 0.1. The histogram is expected to have local maxima corresponding to CN values present in a given sample. Let *n* be the total number of the local maxima and let *T*_*k*_ be the positions of the maxima (*k* = 1,…, *n*). For a given pair of parameters *α* and *β*, for each maximum *k* we assign an integer CN value *c*_*k*_ which minimizes the absolute difference |log  (*T*_*k*_) − log  (*S*(*c*_*k*_))| where *S*(*c*) is given by (7). Then we calculate the following sum:

(9) $$E_{1}\left(\alpha ,\beta \right)=\underset{k}{\sum }H\left(T_{k}\right){\left(\log\;\;\left(T_{k}\right)-\log\;\;\left(S\left(c_{k}\right)\right)\right)}^{2}.$$

This sum measures how well a given pair of parameters *α* and *β* fits the positions of all the maxima *T*_*k*_. In order to find the best parameters *α*_0_ and *β*_0_, we minimize *E*_1_(*α*, *β*) by direct enumeration of all pairs of *α* and *β* with *α* within the interval (0,1) and *β* within the interval (0.5,2). Unfortunately, this minimization is not always able to distinguish between the correct solution and incorrect ones. In order to avoid incorrect solutions, before optimization over parameters *α* and *β* we assign CN *c*_*k*_ to some of the maxima *T*_*k*_ according to the following rules.

1. If there is only one maximum (*n* = 1), then *c*_1_ = 2.
2. If *n* > 1 and the first maximum has SNP genotyping call rate below 60%, then *c*_1_ = 0.
3. If *n* > 1 and the first maximum has SNP heterozygosity below 3%, then *c*_1_ = 1.
4. If *n* > 2, *c*_1_ = 0 and the second maximum has SNP heterozygosity below 3%, then *c*_1_ = 1.
5. If *n* > 1 and the highest maximum is at least two times higher than the second highest maximum, then the highest maximum is assigned CN = 2.

Rule (2) is based on the assumption that the SNP genotyping call rate should be low in the regions with CN = 0. Rules (3) and (4) are based on the assumption that SNP heterozygosity should be low in the regions with CN = 1. Both assumptions are valid for cell lines and tumors with low contamination with noncancerous DNA. Rules (1) and (5) are based on the assumption that the dominating CN equals 2.

### 3.4. Hidden Markov Model

The likelihood that SNPs have copy numbers *c*_*i*_ can be written as

(10) $$\begin{array}{cc} L\left\{c_{i}\right\} & =\overset{}{\underset{i}{\prod }}\exp\;\;\left(-\frac{{\left(\log\;\;\left(S_{i}\right)-\log\;\;\left(S\left(c_{i}\right)\right)\right)}^{2}}{\left(2\sigma^{2}\right)}\right)\\ & \;\times \overset{}{\underset{i}{\prod }}\left(\Delta \left(c_{i+1},c_{i}\right)+\gamma \left(1-\Delta \left(c_{i+1},c_{i}\right)\right)\right). \end{array}$$

Here the first product is taken over all SNPs, and the normal distribution is assumed for deviation of the natural logarithm of the actual SNP signal *S*_*i*_ forms the natural logarithm of the expected signal *S*(*c*_*i*_). In addition, the deviations are assumed to be independent and to have the same standard deviation *σ*. The second product is taken over all pairs of adjacent SNPs, Δ(*c*_*i*+1_, *c*_*i*_) = 1 if *c*_*i*+1_ = *c*_*i*_ and Δ(*c*_*i*+1_, *c*_*i*_) = 0 otherwise, and *γ* is the probability that the adjacent SNPs have different CNs. The most likely CN solution is the one that maximizes the likelihood *L*{*c*_*i*_}. The most likely solution depends both on *σ* and *γ* or, more precisely, on the value of −*σ*^2^log  (*γ*).

The likelihood defines Hidden Markov Model (HMM) with the states being CNs of individual SNPs [18, 19]. Therefore one can use forward-backward procedure [19] to find the maximum likelihood state. In order to make the model computationally tractable, we limit the largest possible CN to be 70. Another simplification is related to the fact that individual chromosomes are independent and, therefore, the maximization of the likelihood is performed separately for all chromosomes. First, we find the maximum likelihood state using values for *α* and *β*  obtained by minimization of the expression (9). Then we minimize over *α* and *β* the following sum:

(11) $$E_{2}\left(\alpha ,\beta \right)=\left(\frac{1}{N}\right)\underset{i}{\sum }{\left(\log\;\;\left(S_{i}\right)-\log\;\;\left(S\left(c_{i}\right)\right)\right)}^{2}.$$

Then we find the maximum likelihood state using values for *α* and *β* obtained by minimization of the expression (11). We repeat this iteration procedure until it converges as defined by change in *α* upon one iteration being less than 3% and change in *β* being less than 0.1%. Formula (11) also provides the estimate of the variance *σ*^2^ in expression (10).

### 3.5. Analysis of 48 Normal Cell Lines

First we have analyzed the data for 48 normal cell lines provided by Affymetrix. One of the advantages of this analysis is that the samples were collected from families (triplets), allowing us to test our algorithm; germ line deletions and amplifications should be inherited from parents. In addition, CN changes are expected to be relatively uncommon. Using this dataset we have optimized parameter *γ* in formula (10) to reduce the number of false positive and false negative CN changes: log (*γ*) = 54. We have found multiple amplifications with CN up to five and heterozygous deletions (CN = 1) as well as a few very small homozygous deletions. These observations are consistent with published results [20]. As expected, we have found CN = 1 for X chromosome for all males. Moreover, for all males we have observed CN = 2 for the pseudoautosomal regions. 

**Figure 2:**
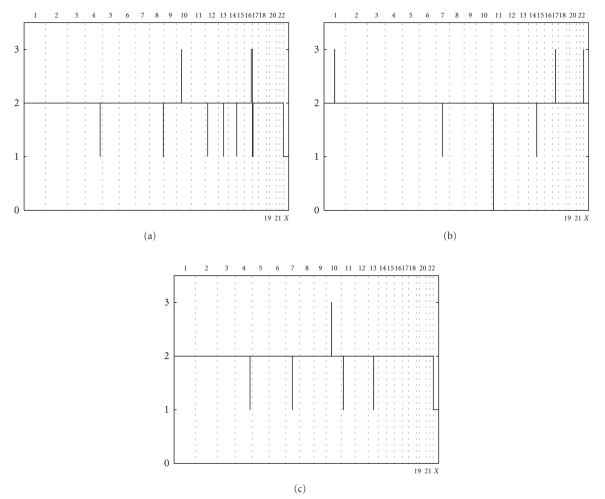
Results of CN analysis for noncancerous cell lines from nuclear family: NA12056 from the father (a), NA12057 from the mother (b), and NA10851 from the son (c).

Results of the analysis of one of the triplets (NA12056 from the father, NA12057 from the mother, and NA10851 from the son) are shown on Figure 2. All deletions and amplifications in NA10851 are, as expected, inherited from the parents: heterozygous deletion on chromosome 4 from the father, heterozygous deletion on chromosome 7 from the mother, amplification to CN = 3 on chromosome 10 from the father, heterozygous deletion on chromosome 11 from the mother (who actually has a homozygous deletion of this region), and heterozygous deletion on chromosome 13 from the father. 

Similar analysis of the other triplets revealed some incompatibilities between CN alterations in children and their parents. Some of these incompatibilities are caused by true somatic CN alterations in the samples as was reported previously [20]. Other incompatibilities are caused by the algorithm either missing some real alterations (false negatives) or reporting some unreal alterations (false positives). We found that the rate of false alterations is high for alterations encompassing only a few SNPs. Starting from seven SNPs the rate of false CN alterations is below 5%.

### 3.6. Analysis of 178 Cancer Cell Lines

**Figure 3:**
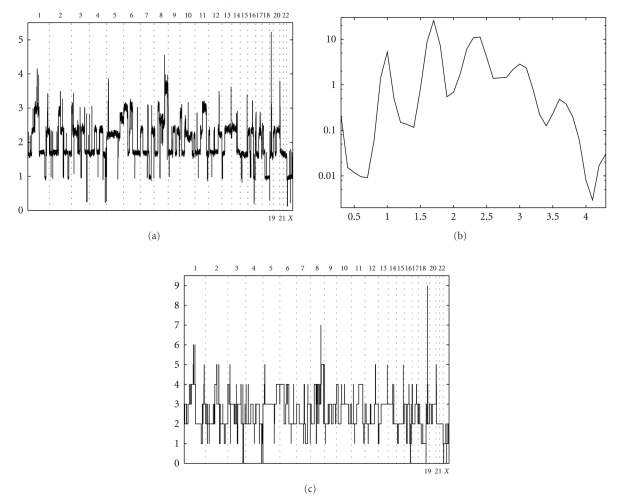
(a) Signal intensity of SNPs (*y* axis) for ovarian cancer cell line OVCAR8 before adjustment on genome size. (b) Fraction of SNPs (*x* axis) with a certain signal intensity (*y* axis) for ovarian cancer cell line OVCAR8 (see (a)). (c) CN solution for ovarian cancer cell line OVCAR8 after adjustment on genome size (*k* = 1.167).

As an example we present here the analysis of the ovarian cancer cell line OVCAR8. Smoothed signals *T*_*i*_ are shown in Figure 3(a) with the corresponding histogram shown in Figure 3(b). There are seven maxima (*n* = 7) in the histogram. Table 2 lists call rates and heterozygosities for SNPs within the seven maxima as well as heights *H*(*T*_*k*_) of the maxima. SNPs within the first maximum have low call rate and thus are assigned CN = 0. SNPs within the second peak have low heterogeneity and thus are likely to correspond to CN = 1. The third peak has to correspond to CN = 2, which is supported by the fact that this peak is the highest. Since this is a cell line, we assume that *α* = 0. Minimization of *E*_1_(*α* = 0, *β*) over *β* gives *β* = 1.18. After adjustment it becomes clear that the fourth peak corresponds to CN = 3, the fifth to CN = 4, the sixth to CN = 5, and the seventh to CN = 6. Running HMM refines the estimate for *β* = 1.17. The final result of CN analysis for this cell line is presented on Figure 3(c). Homozygous deletions of several genes are observed including EVI1 and WWOX.

We have analyzed 178 different cancer cell lines (44 breast, 35 colon, 19 brain, 14 ovarian, 14 lung, 13 melanoma, 11 leukemia, seven pancreatic, six bladder, three kidney, two uterus, two testicular, two prostate, two lymphoma, one thyroid, one salivary gland, one retina, and one plasmacytoma). The number of CN variations in these cell lines varied from two to 369 (the median value being 94). We have observed 510 homozygous deletions encompassing seven or more SNPs. Homozygous deletions, which were observed in at least five different cancer cell lines, are shown in Table 3. Some of these deletions encompass well-known tumor suppressor genes such as p16 and PTEN. Others might encompass unknown tumor suppressor genes, and some of these deletions might simply happen within fragile parts of the genome. We have also observed 580 amplifications with CN of at least 10. Those amplifications, which were observed in at least five different cancer cell lines, are shown in Table 4. Some of these amplifications encompass well-known oncogenes such as MYC and ERBB2. Others might encompass oncogenes which are not yet known.

### 3.7. Adjustment on Contamination with Benign Tissue in Tumor Samples

 In order to check how well our method estimates the degree of contamination *α* of tumors with benign tissue, we artificially contaminated DNA samples of eight cancer cell lines (see Table 5) from different tissues with DNA extracted from CEPH cell line NA12776. All cancer cell lines as well as NA12776 were sampled from female subjects. The DNA samples were quantitated by picogreen three times for higher precision and then combined to give degrees of contamination of the different cancer cell lines with NA12776 DNA between 10% and 80%. After that we ran these mixed samples on Affymetrix microarrays and estimated degrees of contamination using our algorithm. Results of this analysis as well as the mix ratio based on DNA quantitation are presented in Table 5. For the majority of samples the differences between the estimated *α* and the DNA quantitation data were within a few percent. This is quite remarkable, because the observed difference is close to the error rate of the picogreen quantitation. As one could expect, the observed differences tend to be higher for higher contamination levels. 

The cancer cell lines used in the mixing experiment were also run on Affymetrix microarrays without any mixing. Comparison between results of CN analysis of mixed versus nonmixed samples is presented in Table 5. The concordance between CN results is expressed as percent of SNPs with the same CN. With one exception the concordance is above 95% for contamination levels below 80%. While the sensitivity of CN analysis inevitably decreases as contamination with benign tissue increases (and small alterations in CN affecting only a few SNPs become indistinguishable from noise), our algorithm is able to determine the degree of contamination as well as major changes in copy number correctly in strongly contaminated samples. 

**Figure 4:**
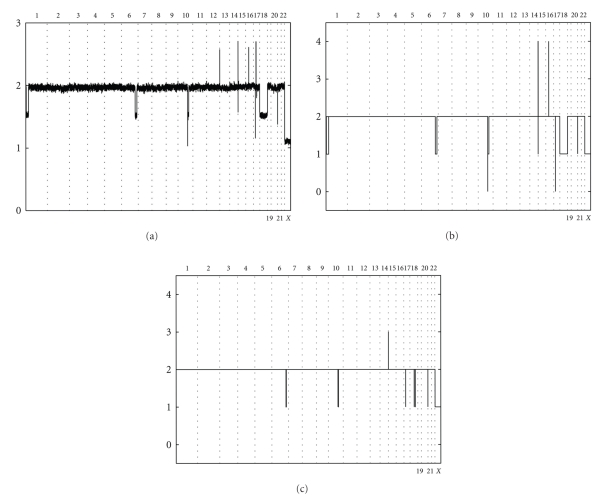
Results of CN analysis of colon tumor sample (collected from male subject) significantly contaminated with benign tissue (~48%). (a) Signal intensity of SNPs (*y* axis) for this sample after adjustment on genome size but before adjustment on contamination with benign tissue. (b) Correct CN solution for this sample after adjustment on both genome size and contamination with benign tissue. (c) Incorrect CN solution for this sample after adjustment on genome size but without adjustment on contamination with benign tissue.

Figure 4 demonstrates the importance of adjustment on the contamination with benign tissue for CN analysis of tumor samples. In Figure 4(a) one can see signal intensities of SNPs for a colon tumor sample. The contamination of this sample with benign tissue, determined by our program, is about 48%. Such strong contamination leads to a dramatic shift of levels of signal intensities for SNPs within regions with different CN values. The right CN solution after adjustment of signal intensity on contamination with benign tissue is presented on Figure 4(b). Because this sample was collected from a male subject, there is only one copy of X chromosome in both tumor and normal cells. As a result, despite contamination the signal intensity for SNPs within the X chromosome (outside the pseudoautosomal regions) is the same as it should be for SNPs within regions with CN = 1 in the absence of any contamination. However, one can see that the signal intensity of SNPs within large heterozygous deletions on chromosomes 1, 6, 10, and 18 is significantly higher than the signal intensity of SNPs within the X chromosome (see Figure 4(a)). SNPs within amplified regions on chromosomes 13, 15, 16, and 17 have signal intensity as if their CNs equal 3. However, after adjustment on contamination it becomes clear that these are actually amplifications to CN = 4. Moreover, SNPs within homozygous deletions on chromosomes 10 and 17 because of the contamination appear to have the same signal intensity as SNPs within the X chromosome. In Figure 4(c) we presented the CN solution, obtained by the HMM algorithm, if adjustment on contamination with benign tissue is not made. All CN variations in this solution have been determined incorrectly (besides single copy of the X chromosome): heterozygous deletions on chromosomes 1 and 15 and amplification on chromosome 16 are lost altogether, positions for the heterozygous deletions on chromosomes 6, 10, 18, and 21 are wrong, homozygous deletions on chromosomes 10 (gene PTEN) and 17 are instead called heterozygous, and, finally, CN = 3 is assigned for the amplification on chromosome 15 instead of CN = 4. Generally we have been able to determine CN values in solid tumors even when the degree of contamination with benign tissue is significant.

### 3.8. Using Agilent 244K Oligonucleotide Arrays for Copy Number Analysis

Agilent 244K oligonucleotide arrays with complete genome coverage are designed for copy number analysis [21, 22]. Instead of SNP probes the array has 60-mer oligonucleotide probes. Our method of analysis of Agilent array data is very similar to the described method of analysis of Affymetrix SNP array data with only two differences. First, we do not need to combine signals from individual probes into an SNP signal; rather we use signal intensities supplied by Agilent software. Second, when we estimate *α* and *β* in (9), we cannot use either SNP call rate or SNP heterozygosity.

## 4. Discussion

In this paper we have presented a new method of CN analysis of the data from Affymetrix 500K GeneChip arrays and Agilent 244K oligonucleotide arrays. The method is designed to determine positions of CN variations as well as to estimate actual CN. In most of the published algorithms, authors attempt to determine position of CN variations and distinguish between deletions and amplifications without determining the actual value of CN [19]. We reason that estimating actual CN is extremely important. For example, it is likely that most CN variations with CN = 1, 3, and 4 are random in the sense that they are not positively selected for during cancer development. On the other hand, homozygous deletions and strong amplifications are most likely to be detected in the regions with genes related to cancer cell survival and growth. 

In order to be able to estimate actual CN within amplified and deleted regions, we have determined how signal intensity from different probes is related to CN, how to take into account change of the genome size in cancer cell lines and solid tumors as the result of somatic alterations, and finally how to incorporate into the analysis contamination of the solid tumors with the benign tissue. HMM has been used to determine exact CN values as well as the positions of the affected regions.

We have tested our method on 48 normal cell lines derived from nuclear families. We have detected a number of germ line CN alterations with proper inheritance from parents to children. We also applied our algorithm to 178 cancer cell lines derived from different tissues. We identified multiple regions with common homozygous deletions and high-level amplifications. As expected some of these regions harbor well-known oncogenes and tumor suppressor genes. Other regions do not encompass well-known cancer-related genes. Then we applied our algorithm to artificial mixes of DNA derived from cancer cell lines with DNA derived from normal cell lines. These mixes model contamination of tumor samples with noncancerous cells. The estimates of the contamination of cancer DNA with normal DNA produced by our algorithm for these mixes matched closely the corresponding experimental estimates using DNA quantitation by picogreen. Comparison between CN alterations detected by our algorithm in the mixed samples versus pure samples showed very high concordance in particular for contamination levels below 50%. Finally, using as an example a colon cancer tumor sample with about 50% contamination with normal DNA, we have demonstrated how erroneous results of CN analysis can be without properly taking into account the effect of the contamination.

There are several limitations to the presented approach. To start with, both Affymetrix and Agilent arrays require a minimum input amount of 500 ng genomic DNA. DNA yields from processed tumor and cell culture samples are highly variable, but typically a minimum of five 10 micron slices of tissue or 10^7^ cells is required to guarantee sufficient DNA yield. While this approach is capable of detecting CN alterations, it cannot detect other aberrations in cell karyotypes such as reciprocal translocations and CN neutral loss-of-heterogeneity regions. This algorithm also cannot distinguish between germline CN variations and somatic CN changes in cancer cell lines and tumors. To be able to do that one has to use normal patient DNA as control. An important limitation of the presented algorithm is related to the assumption that all cancer cells within a cell line or a tumor sample have the same CN profile. This assumption seems to be correct for most of the cell lines and tumor samples we have analyzed. However, some samples (~10%) are clearly heterogeneous; not all the cells in these samples share all CN alterations. For such samples our method produces erroneous results within the regions not shared by all cancer cells. Fortunately, these regions represent only a fraction (~5%) of the genome and, therefore, do not impact significantly the results for the rest of the genome. Another limitation of the algorithm is the assumption of independence of signal noise for closely positioned probes (see (10)). However, it is reasonable to expect that the noise for closely positioned probes should be correlated. We do observe such correlation but the correlation appears to be negligible for Affymetrix 500K SNP arrays and Agilent 244K oligonucleotide arrays. On the other hand, we observed a very strong correlation for adjacent probes on custom Agilent arrays with extremely dense coverage of specific regions (data not shown). This correlation strongly affects the performance of our algorithm and, most likely, other published algorithms; in particular, multiple false CN alterations are being observed. It would be advantageous to modify the presented algorithm to take properly into account this correlation for arrays with dense coverage.

## Figures and Tables

**Table 1:** Median signal intensities calculated using parameters generated in this paper.

Cell line	Median signal	Comment
PC3	0.36	Homozygous deletion of PTEN
Data for 28 male cell lines provided by Affymetrix	1.14	Single copy of X chromosome outside of pseudoautosomal regions
NA04626	2.72	47,XXX
NA01416E	3.41	48,XXXX
NA06061	4.1	49,XXXXX
HTB27	5.31	CN estimated by qPCR for CCND1 is 6.5 ± 1.1
CRL1620	5.79	CN estimated by qPCR for EGFR is 8.6 ± 1.4
CCL251	6.41	CN estimated by qPCR for CCND1 is 10.6 ± 4.4
HTB25	8.22	CN estimated by qPCR for CCND1 is 13.8 ± 1.4
CRL2338	8.82	CN estimated by qPCR for CCND1 is 16.6 ± 0.7
HTB27	9.42	CN estimated by qPCR for ERBB2 is 18.6 ± 2.7
CRL1978	7.95	CN estimated by qPCR for CCND1 is 19.6 ± 6.5
HTB23	11.26	CN estimated by qPCR for CCND1 is 20.6 ± 2.6
HTB19	10.13	CN estimated by qPCR for EGFR is 20.8 ± 2.8
HTB127	13.93	CN estimated by qPCR for CCND1 is 26.1 ± 5.5
CRL2321	14.13	CN estimated by qPCR for CCND1 is 26.6 ± 5.7
HTB41	13.25	CN estimated by qPCR for CCND1 is 46.9 ± 7.1
HTB128	17.92	CN estimated by qPCR for CCND1 is 54.4 ± 14.7
HTB127	21.34	CN estimated by qPCR for ERBB2 is 56.5 ± 3.9

**Table 2:** Heterozygosity and call rate of SNPs within seven maxima of signal intensity for ovarian cancer cell line OVCAR8 (see Figure 3(b)).

Position of a maximum	SNP heterozygosity (%)	Call rate (%)	Height (%)
0.3	9.1	36.7	0.2
1.0	1.2	90.2	5.3
1.7	8	88.1	25.8
2.4	14.2	82	11.2
3.0	19	77.6	2.8
3.6	1	98.4	0.5
4.3	28.9	69.6	0.03

**Table 3:** Frequent homozygous deletions observed in 178 cancer cell lines. Chromosomal positions are based on March 2006 version of the UCSC Human Genome Browser.

Region	Number of observation	Likely tumor suppressor gene
chr2:141238933-141834821	7	LRP1B
chr3:59686057-61195849	29	FHIT
chr4:91615328-92272605	6	
chr6:162091010-162697695	5	PARK2
chr7:38238069-38371496	6	
chr7:141707985-142210594	5	
chr9:19866496-28550130	36	CDKN2A (p16), CDKN2B
chr10:88921717-91164155	6	PTEN
chr14:21159134-22067364	7	
chr16:6161277-7101949	9	
chr16:76814181-77773689	26	WWOX
chr20:14275899-15352425	22	

**Table 4:** Frequent amplifications with CN >9 observed in 178 cancer cell lines. Chromosomal positions are based on March 2006 version of the UCSC Human Genome Browser.

Region	Number of observations	Likely oncogene
chr8:81240524-81974370	5	TPD52
chr8:113091665-114742342	10*	
chr8:128756816-128849113	17	MYC
chr8:142078709-142107781	7**	PTK2
chr11:68619900-69985448	11	CTTN, FGF4, FGF3, FGF19, ORAOV1, CCND1, MYEOV
chr17:34999505-35264341	14	ERBB2
chr17:43477124-44795465	10	
chr17:55677511-61149311	6	APPBP2, PPM1D, BCAS3
chr20:45554593-46054338	5	NCOA3
chr20:55503756-56241375	5	
chr20:57622421-58603945	6	
chr22:19057363-20130955	5	CRKL

*One of these amplifications also involves MYC.

**Two of these amplifications also involve MYC.

**Table 5:** Comparison of the degree of contamination of cancer cell lines with CEPH cell line NA12776 determined using the picogreen quantitation (averaged over three measurements) and the algorithm presented in this paper.

Cancer cell line	Tissue type	Mix ratio from DNA quantitation	Mix ratio from CN analysis	Percent of SNPs with concordant CN values for contaminated versus pure cancer cell lines
HTB19	Breast	10 ± 1%	9.6%	97.1%
HTB76	Ovary	20 ± 4%	18.2%	98.6%
HTB127	Breast	30 ± 4%	33.6%	98.5%
CCL228*	Colon	40 ± 3%	43.4%	95.9%
CCL253*	Colon	50 ± 2%	60.7%	88.2%
HTB119	Lung	60 ± 2%	54.1%	99.8%
HTB9	Urinary bladder	70 ± 2%	74.3%	97.0%
HTB41	Salivary gland	80 ± 1%	84.7%	56.9%

*For these two samples one of the picogreen measurements failed.
